# Tracking protein function with sodium multi quantum spectroscopy in a 3D-tissue culture based on microcavity arrays

**DOI:** 10.1038/s41598-017-04226-2

**Published:** 2017-06-21

**Authors:** Andreas Neubauer, Cordula Nies, Victor D. Schepkin, Ruomin Hu, Matthias Malzacher, Jorge Chacón-Caldera, David Thiele, Eric Gottwald, Lothar R. Schad

**Affiliations:** 1 Computer Assisted Clinical Medicine, Centre for Biomedicine and Medical Technology Mannheim, Heidelberg University, Mannheim, Germany; 2 Institute of Functional Interfaces, Karlsruhe Institute of Technology, Karlsruhe, Germany; 3 CIMAR, National High Magnetic Field Laboratory/FSU, Tallahassee, FL USA; 4 Institute for Biological Interfaces-5, Karlsruhe Institute of Technology, Karlsruhe, Germany

## Abstract

The aim of this study was to observe the effects of strophanthin induced inhibition of the Na-/K-ATPase in liver cells using a magnetic resonance (MR) compatible bioreactor. A microcavity array with a high density three-dimensional cell culture served as a functional magnetic resonance imaging (MRI) phantom for sodium multi quantum (MQ) spectroscopy. Direct contrast enhanced (DCE) MRI revealed the homogenous distribution of biochemical substances inside the bioreactor. NMR experiments using advanced bioreactors have advantages with respect to having full control over a variety of physiological parameters such as temperature, gas composition and fluid flow. Simultaneous detection of single quantum (SQ) and triple quantum (TQ) MR signals improves accuracy and was achieved by application of a pulse sequence with a time proportional phase increment (TQTPPI). The time course of the Na-/K-ATPase inhibition in the cell culture was demonstrated by the corresponding alterations of sodium TQ/SQ MR signals.

## Introduction

Assessment of protein function has long been an overarching goal in biological sciences. Of particular interest ﻿is the function of transmembrane proteins and its impact on cellular regulation. Invasive recording and imaging techniques such as patch clamp^1, 2^ or fluorescence imaging^3^ have been proposed to access protein function by measuring ionic currents through the cell’s plasma membrane with ultra-high sensitivity. Despite the outstanding sensitivity of these methods, their invasiveness hampers long-term monitoring of living cells. Fluorescence imaging requires the loading of the cells with a dye, which often affects cell physiology or even leads to cell death. Patch clamp techniques enable the recording of currents through single channels but are highly sensitive towards operator errors. In this study we describe for the first time, a novel, innovative method to record protein activity in an actively perfused 3D-bioreactor culture noninvasively by means of multi quantum (MQ) spectroscopy.

Noninvasive nuclear magnetic resonance (NMR) techniques can give insight into the concentration and composition of various substances^4^. Sodium has a nuclear spin of 3/2 and along with the concentration in the human body of 80 mM it is detectable by means of NMR^5^. In the presence of an external magnetic field, the sodium spin can occupy four different energy levels, whereas, the proton spin can occupy just two different energy levels. Spin value and the presence of an electrical quadrupolar moment for sodium allow a generation of MQ nuclear resonances which occur with single, double and triple sodium resonance frequency, respectively^6–11^. According to their occurrence with multiple resonance frequencies, these resonances are referred to as single quantum (SQ) double quantum (DQ) and triple quantum (TQ). MQ resonances can be a very sensitive tool to examine interactions of the sodium nucleus with its environment^7, 8, 10–22^.

Pioneering experiments from Hatanaka *et al*. demonstrated the feasibility of MQ NMR by the application of pulsed spectroscopy, laying the foundation of intense studies of quadrupolar nuclei with pulsed NMR spectroscopy^23^. Pulse sequences and hardware tailored for exciting and detecting resonances of sodium nuclei, as well as deviation of specific relaxation times have been presented by several groups^7–13, 20, 24–30^. The behavior of MQ resonances of spin-3/2-nuclei in crystals and dense suspensions with yeast cells was extensively studied by Rooney *et al*.^8, 31, 32^. One of the key findings obtained by Rooney *et al*. was that TQ resonances occur if and only if sodium is interacting with macromolecules such as proteins while SQ resonances are present with and without interaction with such molecules^8^. Schepkin *et al*. observed an increase in the sodium TQ signal in an isolated perfused rat heart during sodium-potassium pump (Na-/K-ATPase) blockage induced by strophanthin^12^. Furthermore, they found a linear correlation between intracellular sodium and the TQ signal. Fonseca *et al*. used sodium MQ spectroscopy to study the binding competition between sodium and lithium in various animal cells. They found out that the intracellular sodium concentration is particularly sensitive to the presence of lithium^33^. Most of these studies record double and triple quantum resonances separate from single quantum resonances using two different pulse sequences. This might hinder a proper quantification, and therefore, limit the quality of assumptions about bound sodium.

In MQ experiments it is advantageous to have a simultaneous observation of SQ and TQ resonances and use the ratio (TQ/SQ), where the SQ resonance serves as an internal reference. Such an approach provides a more accurate detection of the changes in activity of sodium binding proteins such as the Na-/K-ATPase. A simultaneous detection of SQ and TQ signals can be achieved by a﻿ triple quantum time proportional phase increment (TQTPPI). It uses variable increments for pulse phases and inter pulse delays, adjusted to allow specific MQ coherence recording^7, 27^. Recently, the first results of the *in vivo* application of the TQTPPI pulse sequence for sodium and potassium was demonstrated by Schepkin *et al*.^11^. In their work they demonstrated the accuracy of the method and presented a model to estimate extra- and intracellular fractions of bound sodium and potassium in a rat head from the ratio of the TQ/SQ signals. Another interesting result was the observation of a competition for binding sites between sodium and potassium in agarose samples. Agarose samples which contained both sodium and potassium showed a reduced ratio of TQ/SQ for sodium while TQ/SQ for potassium was constant. Therefore, the usage of the TQTPPI sequence and the derivation of TQ/SQ seems the best way to estimate protein binding in living systems.

However, assessment of *in vivo* processes is hampered by several confounding factors including physiological variations of ionic currents or dynamic effects such as perfusion, limiting the temporal and spatial resolution as well as the specificity. Cell suspensions have been previously proposed for an improved control of endogenous factors^32^. Nevertheless, several aspects of cell suspensions are not *in vivo* mimetic including perfusion and cell signaling, limiting the scope of previously obtained findings.

Gottwald *et al*. recently introduced a magnetic resonance (MR) compatible bioreactor for perfusion experiments with densely packed three dimensional cell cultures in a magnetic resonance imaging (MRI) system^34^. The dynamic MRI studies conducted by this group have proven the excellent filling and wash out characteristics of the bioreactor to be independent of flow rates, making it highly suitable for the homogenous distribution of biochemical substances.

In this study we used an improved bioreactor version to investigate the response of three dimensional cell cultures to a strophanthin induced inhibition of the Na-/K-ATPase by means of MQ sodium spectroscopy in order to elucidate the ability of MQ spectroscopy to detect protein activity.

## Material and Methods

### Bioreactor

**Figure 1:**
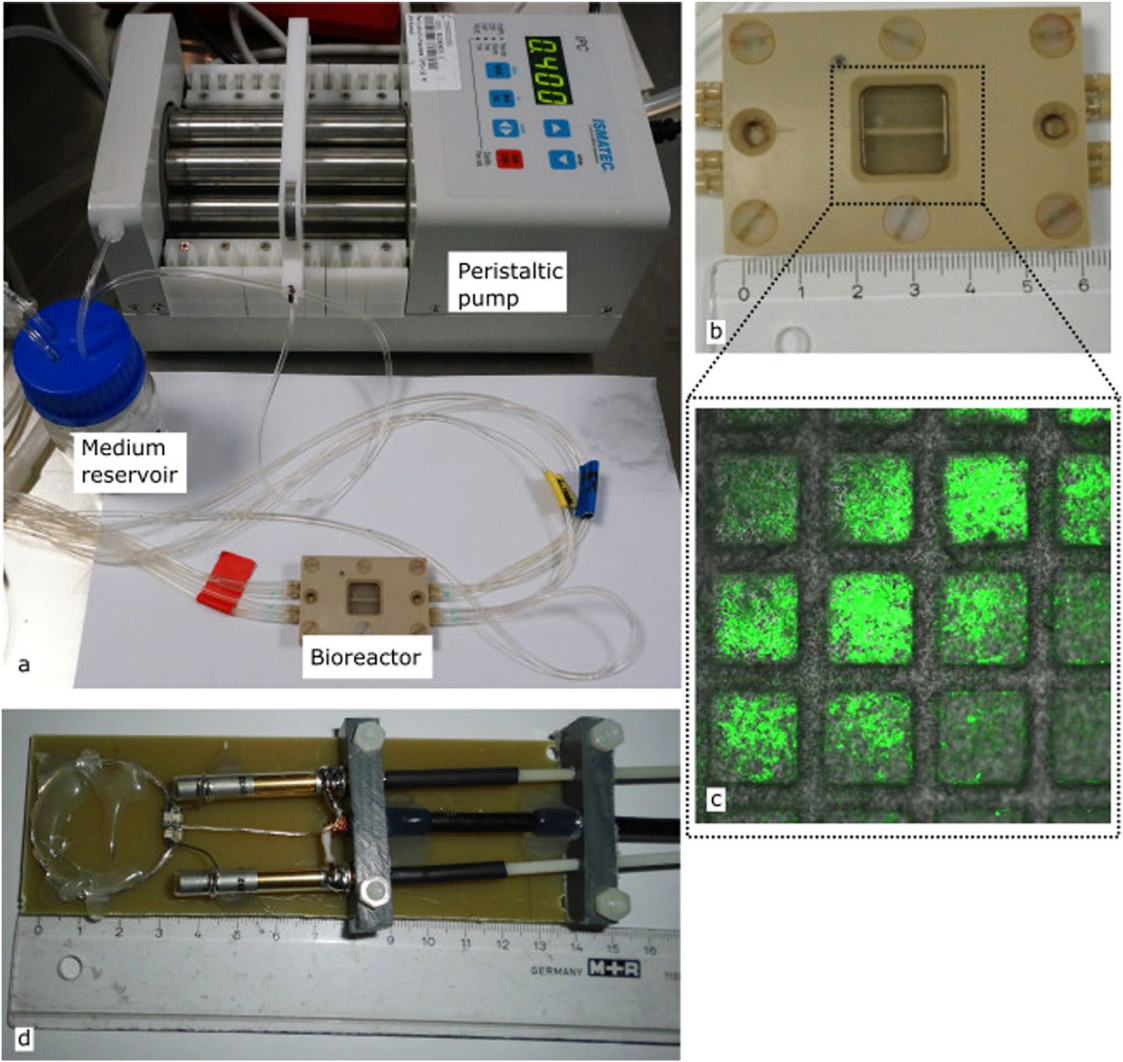
(**a**) Bioreactor connected to the peristaltic pump which produces the pulsatile flow of 400 µl/min and a reservoir for cell culture medium. (**b**) Zoomed image of the bioreactor. The dotted line shows the area where the microcavity array is hosted in the reactor. (**c**) Fluorescence image of the microcavity array with cultured cells. The green color indicates living cells. (**d**) Custom built sodium surface coil.

Experiments were conducted using a MRI-compatible bioreactor containing a 3D culture in microcavity arrays that can be actively perfused as has been described earlier^35^. Further components of the setup are a reservoir for cell culture medium connected to a peristaltic pump and a gas mixing station. The bioreactor, connected to the perfusion pump and the medium reservoir, is shown in Fig. 1a. This setup ensures that during the whole experiment the cells can be supplied with cell culture medium at normoxic or otherwise required conditions. In contrast to the reactor which was previously used by Gottwald *et al*.^34^, this reactor has two compartments which can be independently perfused. Experiments with different perfusion conditions for each compartment are possible with this reactor type. A zoomed image of the bioreactor is depicted in Fig. 1b. Constant temperature of 37 °C inside the reactor was achieved by placing the reactor on a pre-heated animal bed. Regular temperature checks with an infrared thermometer (IR-SCAN-350RH, Voltcraft, Hirschau, Germany) were performed after removal of the reactor from the scanners’ isocenter.

### Cell culture

For all experiments the hepatoma cell line Hep G2 (ATCC, HB-8065, Manassas, VA, USA) was used. Passaging and processing the cells for the bioreactor experiments was done according to previous works^34–36^. The main steps to inoculate the cells into the microcavities were as follows: the microcavity array was placed into a 6 cm petri dish and a drop of 150 µl cell culture medium containing approximately 6 × 10^6^ cells was placed on top of the microcavity array. During the following two hour incubation period under standard cell culture conditions, the cells sedimented into the microcavities. Afterwards, 5 ml cell culture medium was added and the arrays were incubated for two more days leading to tissue-like three dimensional cell cultures. Fluorescence microscopy was performed to proof the viability of the cells after inoculation of the array (Fig. 1c).

Two identical laboratory bottles with 100 ml volume were put into a warm water bath. For proton measurements, one bottle contained 40 ml cell culture medium while the other contained cell culture medium spiked with 2 mM of the contrast agent gadoterate meglumine (Dotarem Guerbet, Paris, France). In case of the TQTPPI experiments, the medium in the second bottle was spiked with 20 mM strophanthin (Sigma-Aldrich, Munich, Germany). The bottles were connected to a three-way valve allowing switching from normal to spiked culture medium and vice versa. Cell viability during the experimental protocol was tested performing lactate dehydrogenase (LDH) measurements before and after treating the cells with 20 mM strophanthin.

### Magnetic resonance setup

All MR experiments were carried out on a conventional small animal scanner with a magnetic field strength of 9.4 T (Biospec 94/20, Bruker, Ettlingen, Germany) using ParaVision 6.0 software. A combination of a commercial proton volume linear transmit-only resonator and a four element receive-only surface coil, placed directly on the bioreactor, was used for experiments at the proton frequency (ω_0,H_ = 400.3 MHz). The direct contrast enhanced (DCE) proton experiments were used to monitor the time of the bolus to reach the bioreactor and to validate a homogenous distribution of the bolus in both chambers of the reactor.

The bioreactor perfusion kinetics were studied after injecting a 6 ml bolus containing 2 mM Dotarem in the circuit. The arrival as well as the distribution of the bolus in the reactor were tracked at a flow rate of 400 µl/min by use of a three dimensional T_1_ weighted RARE sequence with the following parameters: TR/TE = 900 ms/15 ms, RARE factor = 10, Repetitions = 32, Matrix = 64 × 64 × 64, FoV = 33 × 27 × 10 mm^3^, scan time per image = 3 min 50 s.

For sodium experiments, a custom built single loop sodium transmit-receive surface coil (Fig. 1d) was used. The coil had a diameter of 3 cm and was fully balanced using two trimmer capacitors for tuning it to the sodium resonance frequency (ω_0,Na_ = 105.9 MHz) and matching the coil to 50 Ω inside the bore. Unloaded and loaded Q factors were equal to 398 and 386, respectively. The coil was directly placed on top of the bioreactor. In order to achieve a sufficient magnetic field homogeneity an automated shimming procedure up to the second order was applied using sodium MR signals.

The microcavity array was placed in the bioreactor immediately before each TQTPPI experiment. In a following step all air bubbles were removed from the perfusion system. Each experiment started with the injection of a 6 ml bolus. In case of the control experiment it was a phantom bolus from two bottles with identical cell culture medium without any other substances. In case of Na-/K-ATPase blockage it was a bolus with 20 mM strophanthin. The medium supply was switched from one culture medium to the other via the three-way valve.

The response of the cell culture to the presence of strophanthin was observed during bolus injection and its wash out. A perfusion protocol (flow rate 400 µl/min) containing four different stages was used. During the first stage (15 min) the strophanthin bolus was pumped into the circulation system. Subsequently, constant supply of normal cell culture medium was maintained for the next 38 min (stage 2). This time interval was chosen based on the DCE perfusion study the and necessity to provide homogenous distribution of the bolus within the reactor volume. The perfusion pump was switched off for the next 30 min (stage 3). Then, the pump was turned on for more than 57 min (stage 4) to ensure a complete clearance of the strophanthin bolus. The resulting overall experimental time was 140 min.

**Figure 2:**
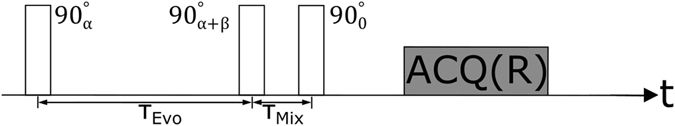
Pulse scheme of the TQTPPI pulse sequence, which consists of three 90° radio frequency excitation pulses with pulse phases α and β. The inter pulse delays are called evolution (T_Evo_) and mixing time (T_Mix_). During the phase cycle α and T_Evo_ are incremented simultaneously while β is altered between 90° and 270°. The receiver for data acquisition is represented by ACQ(R).

MQ spectroscopy was performed using a TQTPPI sequence with three 90° excitation radio frequency (RF) pulses according to the pulse scheme 90° − T_Evo_ − 90° − T_Mix_ − 90° (Fig. 2)^11^. The parameters T_Evo_ and T_Mix_ depict the evolution and mixing time, respectively. A 16 step phase cycle with corresponding time step increments was used to separate SQ and TQ resonances. The contributions from DQ resonances are rather unspecific and their suppression can enhance the quality of the applied fitting routine (see paragraphs below for more details). The proposed phase cycling scheme consists of two phase parameters (α and β), which are applied to the first and second RF pulse. The third RF pulse was applied at a constant phase. During the phase cycle, the parameter β was altered between 90° and 270° after each step while α was incremented by steps of 45° after each second iteration. Along with the phase increment of α, the evolution time was simultaneously incremented with the step width Δ (typically 150 µs). The subsequent steps with different values for β and equal values for α and T_Evo_ were added up to suppress contributions from DQ transitions. The value for T_Mix_ was kept at the minimal value, equal to the pulse length. The length of the excitation RF pulses was dependent on the density of the cell culture, the typical values were found to be between 30 and 70 µs.

Thus, 640 free induction decays (FIDs), were recorded using a TR of 140 ms and 2048 complex data points resulting in an acquisition time of 4 min for each single TQTPPI spectrum. With the proposed sequence parameters 35 TQTPPI spectra were necessary to cover the whole perfusion protocol.

### Data analysis

The phase shifts in the MR signal arising from the receiving electronic were removed. Each first FID in the phase cycle was phase corrected with respect to the acquisition time domain. The same correction factor was applied to every following FID. Subsequently, a Fourier transform (FT) was performed along the acquisition time domain. Real parts of the resulting spectra at the sodium resonance frequency ω_0,Na_ along the evolution time domain were used to construct a TQTPPI-FID. Sodium TQTPPI spectra were obtained by applying a second FT to the constructed TQTPPI-FID. Real parts of all TQTPPI spectra were phase corrected manually and fitted in MATLAB (R2015a, The MathWorks Inc., Natick, MA, USA) using a non-linear least squares fit. The following spectral peak models were used for fitting the SQ and TQ data^7, 27^:

SQ Part:

$${{\rm{S}}}_{{\rm{SQ}}}({{\rm{S}}}_{0,{\rm{SQ}}},{{\rm{\lambda }}}_{{\rm{SQ}}},{{\rm{\omega }}}_{{\rm{SQ}}},{{\rm{\omega }}}_{0,{\rm{SQ}}},{{\rm{C}}}_{{\rm{SQ}}})={{\rm{S}}}_{0,{\rm{SQ}}}\frac{{{\rm{\lambda }}}_{{\rm{SQ}}}}{{{\rm{\lambda }}}_{{\rm{SQ}}}^{2}{({{\rm{\omega }}}_{0,{\rm{SQ}}}-{{\rm{\omega }}}_{{\rm{SQ}}})}^{2}}+{{\rm{C}}}_{{\rm{SQ}}}$$

TQ Part:

$$\begin{array}{rcl}{{\rm{S}}}_{{\rm{TQ}}}({{\rm{S}}}_{0,{\rm{TQ}}},{{\rm{\lambda }}}_{{\rm{TQ}},1},{{\rm{\lambda }}}_{{\rm{TQ}},2},{{\rm{\omega }}}_{{\rm{TQ}}},{{\rm{\omega }}}_{{\rm{SQ}}},{{\rm{C}}}_{{\rm{TQ}}}) & = & {{\rm{S}}}_{0,{\rm{TQ}}}(\frac{{{\rm{\lambda }}}_{{\rm{TQ}},1}}{{{\rm{\lambda }}}_{{\rm{TQ}},1}^{2}{(3{{\rm{\omega }}}_{0,{\rm{SQ}}}-{{\rm{\omega }}}_{{\rm{TQ}}})}^{2}}\\  &  & -\frac{{{\rm{\lambda }}}_{{\rm{TQ}},2}}{{{\rm{\lambda }}}_{{\rm{TQ}},2}^{2}{(3{{\rm{\omega }}}_{0,{\rm{SQ}}}-{{\rm{\omega }}}_{{\rm{TQ}}})}^{2}})+{{\rm{C}}}_{{\rm{TQ}}}\end{array}$$

S_0,SQ_, S_0,TQ_, ω_SQ_ and ω_TQ_ depict the signal amplitudes and frequencies of the different resonances, respectively. The full width at half maximum (FWHM) for the single Lorentzian SQ resonance is represented by λ_SQ_. TQ spectral peaks were assumed to consist of two peaks with different values λ_TQ,1_ and λ_TQ,2_ for FWHM^7, 27^. Prior to fitting, expected SQ (ω_0,SQ_) and TQ (ω_0,TQ_) resonance frequencies were estimated according to the published procedure^27^. Constants C_SQ_ and C_TQ_ were added to compensate for DC offsets during acquisition. Areas under the SQ and TQ peaks were calculated by numeric integration of the fitted curves and used to calculate the relative TQ intensity (TQ/SQ).

Correlation analysis was also performed in MATLAB using its built in corr() function to evaluate correlation coefficients (R) and significance levels (p) values.

## Results

### Perfusion kinetics

**Figure 3:**
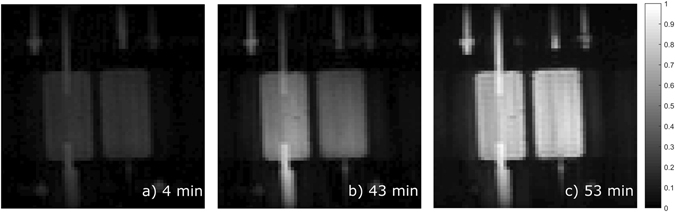
(**a**) The image was recorded 4 min after the start of the experiment, meaning that the bolus with contrast agent was currently injected into the circulation system. (**b**) 43 min after bolus injection the left compartment already shows a homogenous distribution of the contrast agent. (**c**) A homogenous distribution of the contrast agent in both compartments is reached 53 min after bolus injection.

Three coronal T_1_-weighted MR images of the bioreactor’s inner chambers, normalized to the maximum intensity after bolus (2 mM Dotarem) injection are shown in Fig. 3. The perfusion of both compartments can be observed, with the left compartment depicting slightly shorter perfusion times. Homogeneous distribution in both compartments is achieved 53 min after the start of the experiment (Fig. 3c).

### Response of cells to control conditions

**Figure 4:**
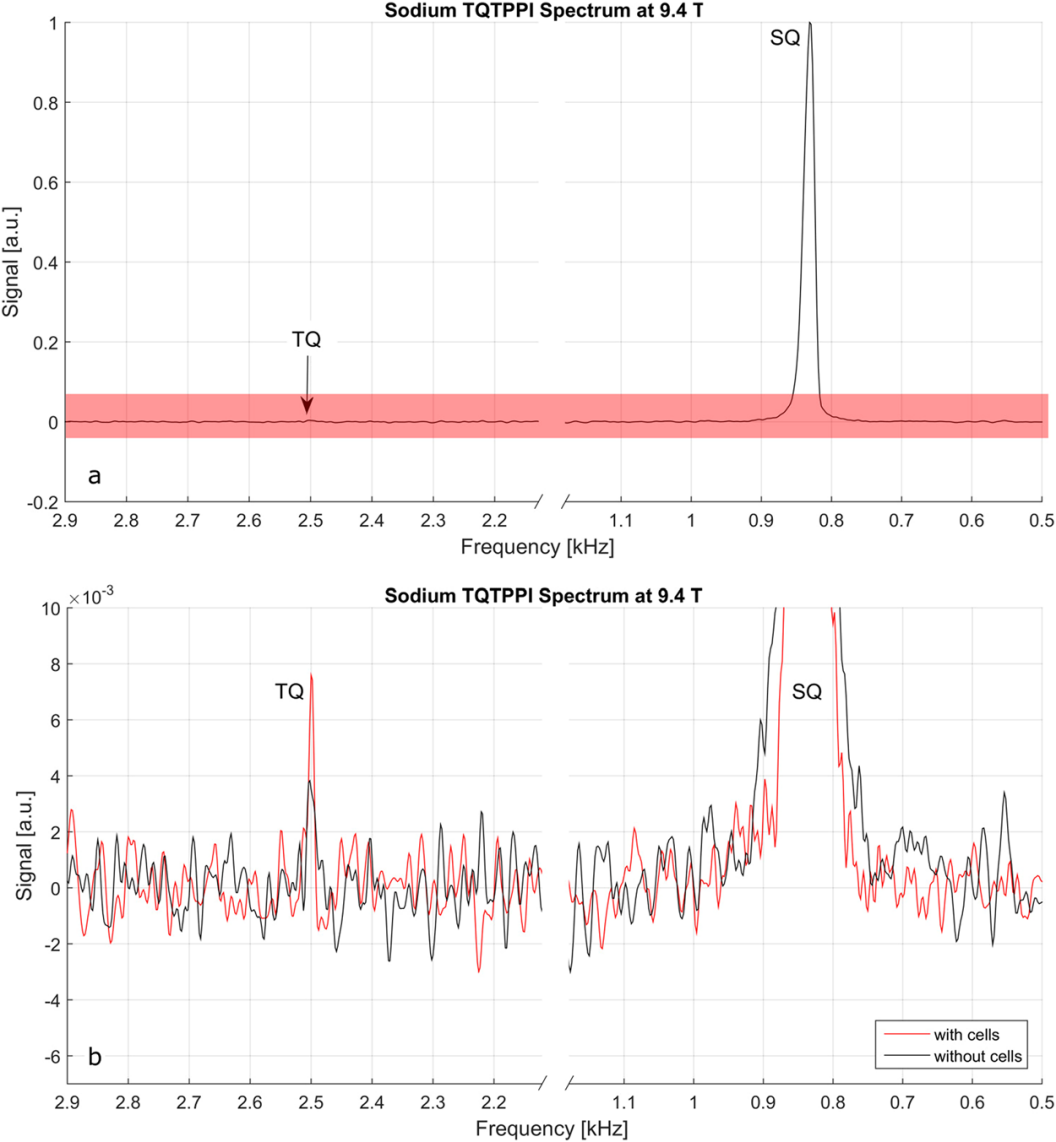
(**a**) Sodium TQTPPI spectrum without cells in full height. The single quantum (SQ) resonance dominates the spectrum. There seems to be no triple quantum contribution. (**b**) Zoomed section of the TQTPPI spectrum (depicted by the red bar in (**a**)). Without cells (black line) a very weak TQ contribution can be observed. Living cells (red line) lead to a markedly higher TQ contribution.

TQTPPI spectroscopy of the baseline medium without cells in the bioreactor shows a well delineated peak of the SQ resonance at 0.8 kHz (Fig. 4a). The zoomed section of the spectrum (red bar in Fig. 4a) shows a slight signal at the TQ frequency of 2.4 kHz (Fig. 4b black line). Substantially increased TQ contribution is present if living cells are inserted in the reactor (Fig. 4b red line). LDH measurements revealed a survival rate of >90% after a perfusion protocol with control medium or strophanthin.

**Figure 5:**
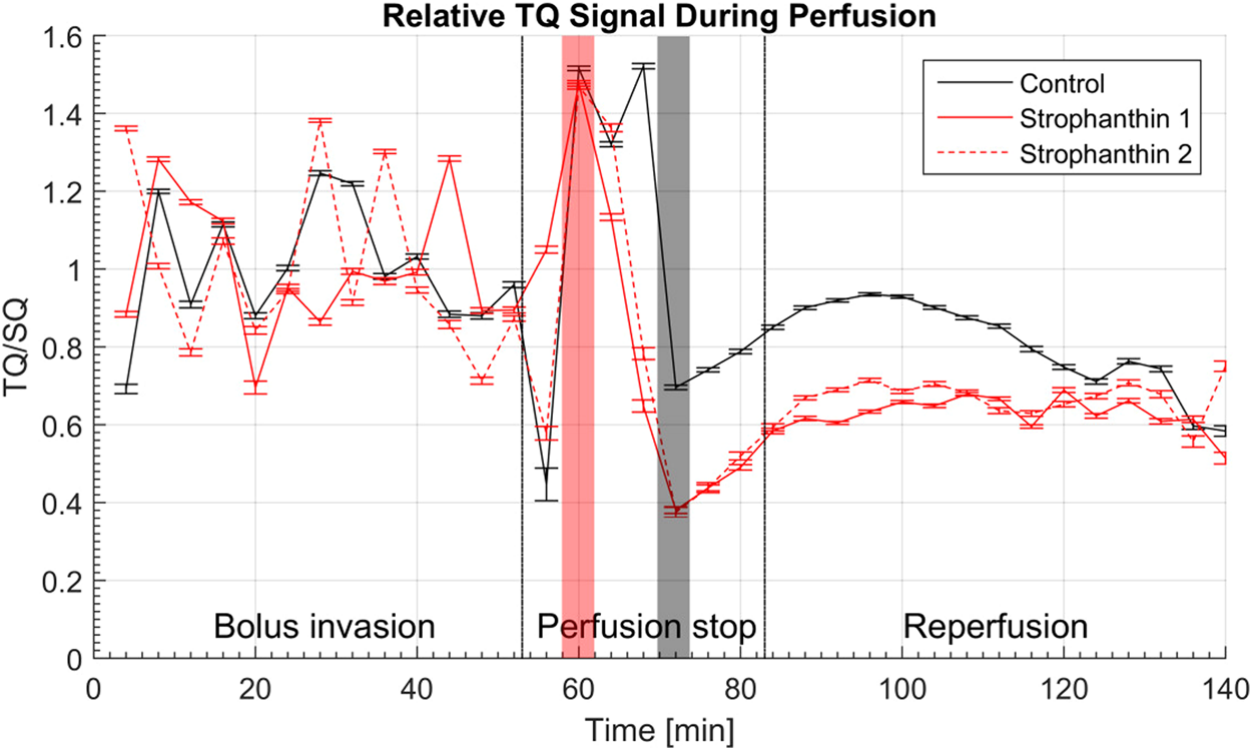
Time course of TQ/SQ during perfusion protocol. The plot is separated into bolus invasion, perfusion stop and reperfusion. Additionally, the data have been normalized to the mean value of TQ/SQ intensity during bolus invasion. The black solid line represents data of the control experiment. Strophanthin experiments are depicted with the red lines. All time courses show an oscillatory behavior prior to perfusion interruption. At 60 min all curves reach their maximum intensity (first red bar). After a drop in intensity at 72 min all curves reach their minimum value (second red bar). Curves recorded from strophanthin induced experiments show a markedly lower drop compared to the control experiment. This is followed by a relaxation period which seems not to be affected by reestablishing perfusion. It can be seen that the difference in signal loss between bolus invasion and reperfusion period is more pronounced in the strophanthin curves than in the control experiment. Oscillations do not occur during perfusion interruption and reperfusion.

In the time course of normal cell culture medium perfusion, oscillations for TQ/SQ were normalized to the mean value and can be presented as 1.00 ± 0.16. It is clearly observed during bolus invasion (Fig. 5 black line). During perfusion interruption, TQ/SQ increases, followed by a drop of the ratio to 0.70 ± 0.01 that recovered during the perfusion pause and does not show a major impact of the reperfusion onwards. The mean value TQ/SQ during the reperfusion period was 0.81 ± 0.11. A relative reduction of the ratio of 19.30 ± 0.14% compared with the baseline signal during initial perfusion was observed during reperfusion.

### Response of cells to strophanthin

The time course for cell cultures treated with strophanthin (Fig. 5 red lines) also shows oscillations of the TQ/SQ ratio during bolus invasion with mean values of 1.00 ± 0.17 and 1.00 ± 0.22, respectively. The stop-flow perfusion led to an increase of TQ/SQ. A markedly lower drop of both curves to 0.38 ± 0.01 at an earlier time point, relative to the perfusion stop, can be observed. In the presence of strophanthin the mean values while washing out the bolus led to TQ/SQ intensities of 0.63 ± 0.04 and 0.67 ± 0.05, respectively. Accordingly, the TQ/SQ ratio reductions are 37.37 ± 0.22% and 33.18 ± 0.18% relative to the bolus invasion period.

Owing to the oscillatory behavior of the TQ/SQ before perfusion interruption, only time points from the first occurring maximum (indicated by the red bar in Fig. 5) to the end of the experiment were used for statistical analysis. Although, the two strophanthin perfusion experiments were recorded from two separate cell cultures, excellent consistency is observed. Correlation analysis between the control experiment and strophanthin induced inhibition experiments led to R-values of 0.76 and 0.73, respectively. Comparison of the strophanthin curves to each other led to R = 0.95. All obtained values for p fulfilled the condition p < 0.01.

## Discussion

In this study, we demonstrated for the first time the ability to monitor the changes in sodium activity in a tissue-like 3D-cell culture system using MQ sodium NMR. Inhibition of the sodium potassium pump by 20 mM strophanthin led to a substantially decreased TQ/SQ signal ratio. This result thus paves the way for non-invasive MQ-NMR based cell culture experiments as well as the monitoring of impaired sodium kinetics prior to cell death *in vivo*.

### Perfusion kinetics and cell viability

Dynamic contrast enhanced MRI measurements revealed homogeneous flow profiles in the bioreactor. Distribution kinetics are comparable to the one chamber system previously presented by Gottwald *et al*.^34^ and have also been shown to be well suited for homogenous supply of medium to densely packed three dimensional cell cultures. At a flow rate of 400 µl/min homogenous distribution of a bolus was achieved 10 min after the bolus entered the bioreactor. Taking into account that most applications require a constant flow through the cell culture, resulting in much higher volumes for the given bolus, the present system seems to be suitable for the medium perfusion study of various cell cultures in an MRI system^37, 38^.

The main goal of the study was to examine the TQTPPI signal of sodium generated by the interaction of sodium ions with proteins during inhibition of the Na-/K-ATPase. Prior to MR experiments we checked the cell viability during the whole experiment by performing LDH tests. As a consequence of the LDH tests it is ensured that signal changes can be linked to the reaction of cells to strophanthin and not to the acute cell death.

### Control conditions

Our results demonstrate the presence of weak TQ resonance even in the absence of living cells. This is well in line with previous findings obtained by Rooney *et al*.^8^, and is most likely due to the interaction of sodium ions with macromolecules in the cell culture medium. Additional interactions with the bioreactor material might also contribute to this small resonance. However, this residual TQ resonance did not exceed the 3σ limit and can be considered as insignificant. A pronounced TQ resonance in the cell culture experiments leads to the conclusion that a significant TQ resonance is clearly related to the presence of living cells. The volume fraction of the cell culture is approximately 1% of the whole volume under investigation; thus, it proves the TQTPPI sequence is a very sensitive tool. An increase of the microcavity array dimensions, a design of the reactor capable to house more cultures or application of phased-array RF microcoils^39^ will help to further enhance the required MR signal. Nonetheless, due to the relation of the TQ signal to the presence of living cells, sodium TQ signals from the cell culture detected by TQTPPI are highly specific with respect to cell physiological processes.

In our experiments we used the sodium SQ MR signal as a reference in order to quantify the bound sodium, which is represented by the TQ signal. However, alterations in the SQ signal itself might also influence values for TQ/SQ ratio. During our experiments we did not observe major alterations in the SQ signal. Thus, variations in TQ/SQ arising exclusively from SQ signal alterations can be considered small compared to the variations of the TQ signal.

During bolus invasion the TQ/SQ signal displayed an oscillatory behavior. This might be related to the pulsatile flow provided by the peristaltic pump. During this period the cells are not yet disturbed by any external intervention and adjusting their protein activity to the pulsatile flow could have a measurable impact.

The steep signal increase in the control experiment without strophanthin, after interruption of the perfusion, is likely to be explained by the decrease in oxygen consumption of the cells. As there is no more medium flow, the remaining medium becomes hypoxic, leading to a reduced activity of the Na-/K-ATPase and therefore to an influx of sodium ions. Increasing of intracellular sodium concentration leads to an increased number of sodium ions interacting with intracellular proteins and, thus, to a higher TQ signal. This is in a good agreement with the results obtained by Schepkin *et al*.^11, 12^. Sodium ions passing through ion channels might also contribute to the TQ signal during that stage. Furthermore, sodium influx alters the osmotic pressure and is, consequently, accompanied by an influx of water into intracellular space, resulting in cell swelling. As soon as a new dynamic equilibrium, including altered sodium concentrations for intra- and extracellular spaces, is established, the overall protein interaction with sodium is reduced based on a higher intracellular space and increased amount of water diluting intracellular protein density. This can be seen by the drop in the TQ/SQ ratio at 70 min. It was also shown by Nakao and Gadsby that the Na-/K-ATPase activity in guinea pig ventricular myocytes is increased if the membrane potential is altered^40, 41^, which might be extrapolated to the present Hep G2 cell line. Based on the findings from Nakao and Gadsby we draw the conclusion that the activity of proteins, not yet affected by hypoxia, increases due to the induced alteration of the transmembrane potential. Accordingly, this leads to an increase of bound sodium and therefore to higher TQ/SQ values after 70 min. Re-establishing the perfusion seems to be ineffective in accelerating the cellular recovery process from hypoxia. In contrast to the behavior during bolus invasion, the TQ/SQ signal did not show any oscillation during the remaining time of the experiment. This insensitivity to a present pulsatile flow might be an indication for ongoing recovery processes. Interestingly, values for TQ/SQ almost reach the level prior to perfusion interruption, indicating good recovery of the cells from hypoxia. After performing the control experiment, a small amount of air bubbles accumulated in the medium chamber. These air bubbles might have entered the reactor during reperfusion, producing a slight inhomogeneity in the magnetic field that led to a signal decrease, which begun after 100 min experiment time.

### Strophanthin

A more pronounced TQ signal drop at an earlier time point during perfusion interruption is observed in the presence of sodium potassium pump inhibition by 20 mM strophanthin. Na-/K-ATPase inhibition leads to a higher water influx resulting in a reduced TQ contribution and therefore to the low values of TQ/SQ ratio. Since sodium gradient regulation is affected by strophanthin, the change in membrane potential can be assumed to be higher than during control conditions. According to Nakao and Gadsby, proteins not affected by strophanthin might increase their activity even more during that period^40, 41^. Nevertheless, the plateau established 10 min after re-establishing perfusion does not reach the level seen in the control experiment, indicating a partial reversibility of the strophanthin induced Na-/K-ATPase inhibition and less bound sodium compared to control conditions. This might be explained as follows. First, it is highly likely that the pump blockage leads to increased intracellular sodium which will be accompanied by a water influx resulting in a higher amount of intracellular water, reducing both, intracellular protein density and intracellular sodium concentration. Thus, we expect less bound intracellular sodium and therefore smaller values for TQ/SQ from the intracellular compartment. This argumentation is well in line with the findings from Schepkin *et al*.^11^ who found that ~53% of the TQ signal can arise from bound sodium in the extracellular space. Since Hep G2 cells hardly form an extracellular matrix in the bioreactor during the time of culture applied, a noticeable TQ contribution from bound extracellular sodium is small. Second, a partial reversibility of the strophanthin induced Na-/K-ATPase inhibition is in good agreement with findings from other groups^41–44^. Accordingly, the TQ signal from sodium binding to Na-/K-pumps can be assumed less compared to control conditions. Together with the consistency of the results, the markedly larger drop of TQ/SQ and the gap between control and strophanthin experiment can be clearly linked to a strophanthin induced reduction of Na-/K-ATPase activity.

### Statistical analysis

The oscillatory behavior of the signal prior to perfusion interruption hinders a proper statistical analysis during this time interval. However, significant changes in the signal can only be assumed to occur when the bolus is distributed in the bioreactor. Our main interest was to analyze sodium signal changes related to the presence of strophanthin. The time interval used for correlation analysis was based on the signal behavior of the control experiment. In order to focus on the impact of strophanthin, the time span beginning from the point where all curves showed maximum signal intensity to the end of the experiment was used for correlation analysis. This revealed an excellent consistency between the strophanthin induced experiments showing the reproducibility of our experiment. Furthermore, a large difference in correlation coefficients between control and strophanthin experiments indicates a statistical significance in our results, emphasizing our hypothesis of a link between TQ/SQ signal and protein activity.

## Conclusion

The technique presented here can be expanded to a vast number of applications. The activity of different proteins stimulated by specific biochemical drugs can be detected by this method. It can be done not only by sodium but also by other MQ signals coming from chlorine and potassium. This approach can be seen as a non-invasive physiological probe which can be easily translated to human MRI systems to study protein activity *in vivo*.

MRI compatible bioreactors allow hosting three dimensional cell cultures with high degree of control over a variety of physiological and physical parameters. A precise control over cell culture during MR experiments can critically enhance the physiological information available from MR data. In the presented study a significant TQ contribution is clearly linked to the presence of living cells. Furthermore, the strophanthin induced Na-/K-ATPase inhibition can be tracked by observing the bound sodium pool with TQ/SQ MR signals derived from sodium TQTPPI spectroscopy. Reductions in TQ/SQ compared to a control experiment are reproducible and statistically significant. Our results are in good agreement with prior data in a rat heart, rat head and recording via patch clamp. The use of this non-invasive technique to investigate protein activity in a variety of tissue-like cell cultures, including for example human tumor cells facilitates a direct comparison between experiments on a cellular level and *in vivo* data recorded on one and the same device.

### Data availability

The data supporting the findings of this study are available from the corresponding author upon reasonable request.

## References

[1] Neher E, Sakmann B (1976). Single-channel currents recorded from membrane of denervated frog muscle fibres. Nature.

[2] Blatz AL, Magleby KL (1983). Voltage-dependent chloride-selective channels of large conductance in cultured rat muscle. Biophys J.

[3] Coons AH, Kaplan MH (1950). Localization of antigen in tissue: improvements in a method for the detection of antigen by means of fluorescent antibody. Jpn J Exp Med.

[4] de Graf, R. A. *In vivo NMR spectroscopy: principles and techniques*. 2nd edn, 570 (John Wiley & Sons 2007).

[5] Haacke, E. M., Brown, R. W., Thompson, M. R. & Ventkatesan, R. 914 (John Wiley & Sons 1999).

[6] Abragam, A. *Principles of nuclear magnetism*. (Oxford University Press, 1961).

[7] van der Maarel JRC (2003). Thermal relaxation and coherence dynamics of spin 3/2. I. Static and fluctuating quadrupolar interactions in the multipole basis. Concept Magn Reson A.

[8] Rooney WD, Springer CS (1991). A comprehensive approach to the analysis and interpretation of the resonances of spins 3/2 from living systems. NMR Biomed.

[9] Jaccard G, Wimperis S, G. B (1986). Multi-quantum NMR spectroscopy of S = 3/2 spins in isotropic phase: a new probe for multiexponential relaxation. J Chem Phys.

[10] Gottwald, E., Neubauer, A. & Schad, L. R. In *Assessment of cellular and organ function and dysfunction using direct and derived MRI* (ed Dr. Christakis Constantinides) Ch. 2, (InTech 2016).

[11] Schepkin VD, Neubauer A, Nagel AM, T. F. B (2017). Comparison of potassium and sodium binding *in vivo* and in agarose samples using TQTPPI pulse sequence. J Magn Reson.

[12] Schepkin VD (1998). Sodium TQF NMR and intracellular sodium in isolated crystalloid perfused rat heart. Magn Reson Med.

[13] Benkhedah N, Bachert P, Nagel AM (2014). Two-pulse biexponential-weighted ^23^Na imaging. J Magn Reson Imaging.

[14] Duvvuri U, Leigh JS, Reddy R (1999). Detection of residual quadrupolar interaction in the human breast *in vivo* using sodium-23 multiple quantum spectroscopy. J Magn Reson Imaging.

[15] Reddy R, Bolinger L, Shinnar M, Noyszewski E, Leigh JS (1995). Detection of residual quadrupolar interaction in human skeletal muscle and brain *in vivo* via multiple quantum filtered sodium NMR spectra. Magn Reson Med.

[16] Hancu I, Boada FE, Shen GX (1999). Three-dimensional triple-quantum-filtered (23) Na imaging of *in vivo* human brain. Magn Reson Med.

[17] Torres AM, Philp DJ, Kemp-Harper R, Garvey C, Kuchel PW (2005). Determination of Na+ binding parameters by relaxation analysis of selected 23Na NMR coherences: RNA, BSA and SDS. Magn Reson Chem.

[18] Colet JM, Bansal N, Malloy CR, Sherry AD (1999). Multiple quantum filtered 23Na NMR spectroscopy of the isolated, perfused rat liver. Magn Reson Med.

[19] Mirkes C (2016). Triple-quantum-filtered sodium imaging at 9.4 Tesla. Magn Reson Med.

[20] Fleysher L (2013). Noninvasive quantification of intracellular sodium in human brain using ultrahigh-field MRI. NMR Biomed.

[21] Petracca M (2016). Brain intra- and extracellular sodium concentration in multiple sclerosis: a 7 T MRI study. Brain.

[22] Madelin G, Lee JS, Regatte RR, A. J (2014). Sodium MRI: methods and applications. Prog Nucl Magn Reson Spectrosc.

[23] Hatanaka H, Terao T, Hashi T (1975). Excitation and detection of coherence between forbidden levels in three-level spin system by multi-step processes. J Phys Soc Jpn.

[24] Bodenhausen G, Vold RL, R. R. V (1980). Multiple quantum spin-echo spectroscopy. J Magn Reson.

[25] Bodenhausen G (1981). Multiple-quantum NMR. Prog Nucl Magn Reson Spectrosc.

[26] van der Maarel JRC (1989). Relaxation of spin S = 3/2 in the doubly rotating tilted frame. J Chem Phys.

[27] van der Maarel JRC (1991). Relaxation of spin quantum number S = 3/2 under multiple-pulse quadrupolar echoes. J Chem Phys.

[28] van der Maarel JRC, Tromp RH, Leyte JC, Hollander JG, C. E (1990). Spin S = 3/2 T1ρ relaxation; the excitation of triple-quantum coherences. Chem Phys Lett.

[29] Malzacher M, Kalayciyan R, Konstandin S, Haneder S, L. R. S (2016). Sodium-23 MRI of whole spine at 3 Tesla using a 5-channel receive-only phased-array and a whole-body transmit resonator. Z Med Phys.

[30] Weingaertner S (2015). Scan time reduction in (23)Na-magnetic resonance imaging using the chemical shift imaging sequence: evaluation of an iterative reconstruction method. Z Med Phys.

[31] Rooney WD, Barbara TM, C. S. SJ (1988). Two-dimensional double-quantum NMR spectroscopy of isolated spin 3/2 systems: 23Na examples. J Am Chem Soc.

[32] Rooney WD, Springer JC (1991). The molecular environment of intracellular sodium: 23Na NMR relaxation. NMR Biomed.

[33] Fonseca CP (2013). 23Na multiple quantum filtered NMR characterisation of Na+ binding and dynamics in animal cells: a comparative study and effect of Na+/Li+ competition. Eur Biophys J.

[34] Gottwald E (2013). Characterization of a chip-based bioreactor for three-dimensional cell cultivation via magnetic resonance imaging. Z Med Phys.

[35] Gottwald E (2007). A chip-based platform for the *in vitro* generation of tissues in three-dimensional organization. Lab Chip.

[36] Altmann B, Giselbrecht S, Weibezahn K-F, Welle A, Gottwald E (2008). The three-dimensional cultivation of the carcinoma cell line HepG2 in a perfused chip system leads to a more differentiated phenotype of the cells compared to monolayer culture. Biomed Mater.

[37] Rieke M, Gottwlad E, Weibezahn K-F, Layer P (2008). Tissue reconstrucion in 3D-spheroids from rodent retina in a motion-free, bioreactor-based microstructure. Lab Chip.

[38] Eschbach E (2005). Microstructured scaffolds for liver tissue culture of high cell density: morphological and biochemical characterization of tissue aggregates. J Cell Biochem.

[39] Goebel K (2015). Phased-array of microcoils allows MR microscopy of *ex vivo* human skin samples at 9.4 T. Skin Res Technol.

[40] Nakao M, Gadsby D (1989). [Na] and [K] dependence of the Na/K pump current-voltage relationship in guinea pig ventricular myocytes. J Gen Physil.

[41] Mitchell TJ, Zugarramurdi C, Olivera JF, Gatto C, Artigas P (2014). Sodium and proton effects on inward proton transport through Na/K pumps. Biophys J.

[42] Albers RW, Koval GJ, G. J. S (1968). Studies on the interaction of ouabain and other cardlo-active steroids with sodium-potassiurn-activated adenosine triphosphatase. Mol Pharmacol.

[43] Sen AK, Tobin T (1969). A cycle for ouabain inhibition of sodium- and potassium-dependent adenosine triphosphatase. J Biol Chem.

[44] Schatzmann HJ (1953). Die Wirkung von Desoxycorticosteron auf den aktiven Kationenaustausch an Rattenblutzellen. Experientia.

